# Experiences of Fertility-Related Anxiety Among Women with Polyendocrine Metabolic Ovarian Syndrome: A Qualitative Study

**DOI:** 10.3390/healthcare14193249

**Published:** 2026-10-01

**Authors:** Selinay Aktaş Demir, Rüveyda Ölmez Yalazı, Destina Gizem Dağcı

**Affiliations:** 1 Department of Midwifery, Faculty of Health Sciences, Istanbul Atlas University, Istanbul 34408, Türkiye; selinay.demir@atlas.edu.tr; 2 Department of Women Health and Gynecologic Nursing, Institute of Graduate Studies, Istanbul University-Cerrahpaşa, Istanbul 34320, Türkiye; 3 Department of Midwifery, Faculty of Health Sciences, Fenerbahçe University, Istanbul 34758, Türkiye; ruveyda.olmez@fbu.edu.tr

**Keywords:** fertility concerns, fertility-related anxiety, polycystic ovary syndrome, polyendocrine metabolic ovarian syndrome, qualitative research, reproductive health, women’s health

## Abstract

**Highlights:**

**What are the main findings?**

- Women described fertility-related anxiety arising from uncertainty about conception, repeated treatment experiences, and social expectations surrounding motherhood.
- Weight-focused healthcare encounters, decisions about disclosing the diagnosis, partner support, and unmet psychological support needs shaped women’s experiences.

**What are the implications of the main findings?**

- Fertility concerns should be discussed from diagnosis onward using balanced, individualized information that does not unnecessarily intensify fear of infertility.
- PMOS care should combine reproductive counselling with psychologically supportive communication and referral according to women’s needs and reproductive intentions.

**Abstract:**

**Background/Objectives:** Polyendocrine metabolic ovarian syndrome (PMOS), formerly known as polycystic ovary syndrome, is a chronic condition with reproductive, metabolic, and psychological consequences. Fertility-related anxiety may be shaped by individual, relational, sociocultural, and healthcare experiences, but these experiences remain insufficiently understood. This study explored fertility-related anxiety and its personal, relational, and healthcare contexts among married women with PMOS who had no children. **Methods:** This qualitative descriptive study included 15 married, childless women recruited through purposive sampling from a private gynecology outpatient clinic in Istanbul, Türkiye. Face-to-face semi-structured interviews were audio-recorded and transcribed verbatim in Turkish. Data were examined using a codebook approach to thematic analysis by three researchers. Reporting followed the Consolidated Criteria for Reporting Qualitative Research. **Results:** The participants’ mean age was 25.5 ± 4.1 years. Nine had previously attempted to conceive, seven had received fertility treatment, and fourteen reported that the diagnosis had affected their thoughts about having children. Five themes were generated: perceptions of the effect of PMOS on fertility; experiences and evaluations regarding treatments; social circle, family, and spousal pressure; expectations from healthcare professionals; and self-management and coping strategies. Participants described anxiety about future conception, cycles of hope and disappointment, social pressure, supportive partner relationships, unmet information and psychological support needs, and efforts to manage their health and emotions. **Conclusions:** Fertility-related anxiety was multidimensional and varied according to women’s individual, relational, and healthcare experiences. Clear individualized fertility information, psychologically supportive communication, and coordinated multidisciplinary care tailored to women’s reproductive intentions and needs may help address these experiences.

## 1. Introduction

Polyendocrine metabolic ovarian syndrome (PMOS), formerly known as polycystic ovary syndrome (PCOS), is a common, chronic, multisystem condition that affects women during the reproductive years and persists into later life [1,2]. The new name was adopted through a multistep global consensus process to reflect the condition’s endocrine, metabolic, and ovarian features and to remove the misleading implication of pathological ovarian cysts [1]. In adults, diagnosis requires at least two of three features after other relevant conditions have been excluded: clinical or biochemical hyperandrogenism, ovulatory dysfunction, and polycystic ovarian morphology on ultrasound. Serum anti-Müllerian hormone may be used instead of ultrasound to define polycystic ovarian morphology in adults, but it should be interpreted within the recommended diagnostic algorithm [3]. Beyond its reproductive manifestations, PMOS is associated with substantial metabolic and psychological health consequences. Women with PMOS have increased risks of insulin resistance, type 2 diabetes, and obesity [2]. They also have a higher prevalence of anxiety and depressive symptoms, while PMOS and its clinical features can adversely affect body image and quality of life [3]. A systematic review and meta-analysis also found higher levels of anxiety and depressive symptoms and lower quality of life among women with PMOS than among women without the condition [4].

PMOS is the most common cause of anovulation globally and a leading cause of infertility [2]. Women with PMOS have reported concerns about whether they would be able to become pregnant, how they should prepare for pregnancy, and what they should do before attempting conception [5]. In the same study, some participants wished to receive fertility-related information at diagnosis or well before considering pregnancy, whereas others preferred to receive it closer to the time of pregnancy planning [5]. In a Turkish study of 140 young adults aged 18–26 years with PMOS, 72.9% expressed concern about future infertility and 61.4% reported sometimes worrying that they would be unable to conceive because of PMOS [6]. A recent qualitative study conducted in Türkiye similarly identified anxiety, sadness, and concerns about infertility as prominent experiences following a PMOS diagnosis [7]. Among married women with PMOS experiencing fertility difficulties, the perceived cultural importance of childbearing and pressure to conceive soon after marriage were associated with higher levels of depression and anxiety [8]. A qualitative study also found that women could experience prolonged anxiety about infertility regardless of symptom severity; for some women, these concerns resulted in pressure to conceive earlier or changes in their life plans [9]. Together, these findings indicate that fertility-related concerns may influence women’s expectations, emotional well-being, and decisions concerning future childbearing [5,6,7,8,9].

Previous research has demonstrated that women with PMOS experience concerns about future fertility, unmet fertility-related information needs, and anxiety associated with receiving the diagnosis [5,6,7,8,9,10,11,12]. Holton et al. [5] qualitatively examined women’s fertility concerns and information needs in Australia, whereas Copp et al. [9] explored women’s experiences of the benefits and harms of receiving a PMOS diagnosis. In Türkiye, Sarı and Güngör Satılmış [6] quantitatively examined concerns about fertility, social appearance, and relationships among young adults with PMOS. Qualitative evidence has also demonstrated insufficient fertility-related guidance, limited professional information, and a perceived lack of empathy in women’s healthcare experiences [10]. Women from an ethnically diverse UK sample additionally described psychological distress, difficulties coping with PMOS, and culturally influenced support needs [11]. Women’s PMOS-related concerns and expectations may also vary according to age and ethnicity, supporting the need for individualized care and educational resources [12]. These studies primarily addressed reproductive concerns as a broad construct that includes beliefs about future fertility, information needs, reproductive intentions, and anticipated infertility. In contrast, in this study, fertility-related anxiety refers to the emotional experience of worry, fear, and uncertainty specifically associated with anticipated or experienced difficulty conceiving. Although Özcan et al. [7] examined women’s emotions following diagnosis, concerns about childbearing, and sources of information in Türkiye, less is known about how this anxiety is experienced specifically among married, childless women with differing histories of attempting conception and receiving fertility treatment. Moreover, previous research has not examined together how fertility-related anxiety is shaped, intensified, or managed across relational, treatment-related, healthcare, and coping contexts. The present qualitative descriptive study therefore focused specifically on fertility-related anxiety among married, childless women diagnosed with PMOS and examined how these experiences were shaped and managed within personal, relational, treatment-related, and healthcare contexts.

## 2. Materials and Methods

### 2.1. Study Design

This study employed a qualitative descriptive design with a codebook approach to thematic analysis to explore fertility-related anxiety among married, childless women diagnosed with PMOS [13]. A qualitative descriptive design was selected to provide a practice-oriented account of participants’ experiences while remaining close to their descriptions. Data were collected through individual semi-structured interviews. The study was reported in accordance with the Consolidated Criteria for Reporting Qualitative Research (COREQ), a 32-item checklist for studies involving interviews and focus groups [14]. The completed COREQ checklist is provided in Supplementary Table S1.

### 2.2. Participants and Setting

Participants were recruited from the gynecology outpatient clinic of a private healthcare institution in Istanbul, Türkiye, between 13 June and 30 July 2026. Women were eligible to participate if they were 18–40 years of age, were married, had no children, had a documented diagnosis of PMOS for at least six months, were able to communicate in Turkish, and provided written consent to participate in an audio-recorded interview. Women were excluded if they had a coexisting reproductive condition that could independently affect fertility or a severe psychiatric or cognitive condition that could prevent meaningful participation in the interview.

Potential participants were identified and approached face-to-face by the researchers at the gynecology outpatient clinic. The researchers assessed eligibility, explained the purpose and procedures of the study, and invited eligible women to participate. Women who expressed an interest were given an opportunity to ask questions before providing written informed consent. Purposive sampling was used to recruit married women with PMOS who had no children and met the study eligibility criteria. Recruitment and preliminary analysis proceeded concurrently. After each interview, the three researchers reviewed the emerging codes and compared them with the cumulative codes and patterns identified in the preceding interviews. For this study, saturation was operationalized as the point at which successive interviews generated no new substantive codes, no new dimensions of existing codes, and no new relationships among the developing patterns that were relevant to the research aim. During the final interviews, participants’ accounts repeatedly confirmed the existing codes and developing themes without requiring substantive revision of the analytic structure. The three researchers reviewed these comparisons together, reached consensus that saturation had been achieved, and concluded recruitment after 15 interviews [15]. All eligible women who were invited agreed to participate, and there were no refusals or withdrawals.

### 2.3. Data Collection

Data were collected by three female researchers with academic and clinical experience in women’s health (S.A.D., R.O.Y. and D.G.D.). None of the researchers was involved in the participants’ clinical care, and none had a prior relationship with the participants. Before the interviews, participants were informed that the interviewers were academic researchers conducting research independently of their clinical care and that participation would not affect the care they received. Interviews were conducted in a private room at the gynecology outpatient clinic to ensure privacy and minimize interruptions. No individuals other than the participant and the researcher conducting the interview were present. Each participant completed one interview, and no repeat interviews were conducted. Each interview lasted approximately 30–40 min and was audio-recorded with the participant’s permission. No field notes were taken.

The semi-structured interview guide was developed by the research team based on the previous literature addressing women’s experiences of PMOS, fertility-related concerns, and information needs [16,17]. It included questions concerning participants’ sociodemographic and clinical characteristics and five open-ended questions addressing their fertility-related experiences associated with PMOS. The five open-ended interview questions are provided in Supplementary Table S2. Before the main data collection, the interview guide was pilot-tested with two women diagnosed with PMOS to assess the clarity, comprehensibility, and flow of the questions. Both women found the questions clear and understandable; therefore, no revisions were required, and the original questions were retained. The two women who participated in the pilot interviews were not included in the main study sample. All audio-recorded interviews were transcribed verbatim in Turkish. Transcripts were not returned to participants for comment or correction. Following completion of the analysis, the themes and selected participant quotations were translated into English and reviewed by the research team for semantic and cultural equivalence. Interviews were conducted shortly after the international name change was published; participants used the term PCOS, which has been retained in the verbatim quotations.

### 2.4. Data Analysis

Data were analyzed using a codebook approach to thematic analysis, structured around the six analytic phases described by Braun and Clarke [18]. A codebook approach was selected to support team-based analysis through a shared coding framework while allowing themes to be developed inductively from the data. This structured and consensus-based approach differs from reflexive thematic analysis [19]. Coding was performed manually without qualitative data analysis software. First, S.A.D., R.O.Y., and D.G.D. independently read the transcripts several times to become familiar with the data and generated initial codes. The three researchers then compared their codes and developed a shared codebook through discussion. The agreed codebook was applied across the dataset, and differences in coding were resolved through consensus. Related codes were grouped into candidate themes, which were subsequently reviewed, refined, defined, and named by the research team. Although the interview topics defined broad areas of inquiry, coding was primarily inductive, and the codes and subthemes were developed from participants’ accounts rather than from a pre-established coding framework. The final themes were refined through comparison across the dataset and selected based on their relevance to the research aim and representation of participants’ experiences. The complete coding tree, showing the progression from initial codes through intermediate categories and subthemes to the final themes, is provided in Supplementary Table S3.

### 2.5. Trustworthiness

Trustworthiness was established according to the criteria of credibility, dependability, confirmability, and transferability described by Lincoln and Guba [20] and Korstjens and Moser [21]. Credibility was supported through independent coding, consensus among the three researchers, external expert review, and the presentation of direct participant quotations. To enhance dependability, the coding process was repeated after two weeks, and each stage of the analysis was documented. Confirmability was supported through reflexive discussions among the three researchers and review of the final themes by two external experts who were not involved in the analysis. Transferability was addressed by providing detailed descriptions of the study setting, participants, and research procedures. Participant checking was not undertaken; participants were not asked to review or provide feedback on the findings. Participant checking was not undertaken because the analysis sought a cross-case thematic interpretation rather than individual endorsement of a single, definitive interpretation. We acknowledge that this precluded direct participant validation. Trustworthiness was instead reinforced through the complementary strategies described above: independent coding and consensus, recoding after two weeks, documentation of each analytic stage, reflexive discussions, external expert review, and direct participant quotations.

### 2.6. Researcher Characteristics and Reflexivity

All three researchers were female academic researchers with clinical and qualitative research experience in women’s health. Two researchers held master’s degrees (MSc), and one researcher held a doctoral degree (PhD). Reflexive discussions were conducted throughout data collection and analysis to maintain awareness of professional assumptions and interpretations [22]. The researchers regularly discussed their interpretations and analytical decisions and reached agreement through collaborative evaluation.

### 2.7. Ethical Considerations

Ethical approval was obtained from the Fenerbahçe University Non-Interventional Clinical Research Ethics Committee (decision no. 72.2026fbu; 9 June 2026). Institutional permission was also obtained from the healthcare institution where the study was conducted. The study was conducted in accordance with the Declaration of Helsinki. Before the interviews, participants received information about the purpose and procedures of the study, the voluntary nature of participation, confidentiality, and their right to withdraw at any time without providing a reason. Written informed consent, including permission for audio recording, was obtained from all participants. Participant identities were protected by replacing personal information with participant codes in transcripts and research reports.

## 3. Results

The study included 15 women with a mean age of 25.5 ± 4.1 years (range: 21–34 years). The mean duration since diagnosis was 5.2 ± 3.8 years (range: 1–14 years). Regarding educational attainment, five participants (33.3%) were high school graduates, seven (46.7%) held a bachelor’s degree, and three (20.0%) held a postgraduate degree. Nine participants (60.0%) were unemployed. All participants were married, and none had children. Nine women (60.0%) had previously attempted to conceive, and seven (46.7%) had received fertility treatment. The most frequently reported PMOS-related symptoms were menstrual irregularity (80.0%), weight gain (53.3%), hirsutism (40.0%), acne (40.0%), and insulin resistance (26.7%). Fourteen participants (93.3%) stated that receiving a PMOS diagnosis had affected their thoughts about having children. Further sociodemographic and clinical characteristics are presented in Table 1.

The thematic analysis generated five themes: (1) perceptions of the effect of PMOS on fertility; (2) experiences and evaluations regarding treatments; (3) social circle, family, and spousal pressure; (4) expectations from healthcare professionals; and (5) self-management and coping strategies. The themes and corresponding subthemes are presented in Table 2. Each theme is described below and supported by direct quotations from the participants. A detailed account of the analytic hierarchy is provided in Supplementary Table S3.

### 3.1. Perceptions of the Effect of PMOS on Fertility

#### 3.1.1. Anxiety and Fear of the Future

Most participants expressed concern about the potential effects of PMOS on their fertility, whereas one participant did not anticipate any fertility-related problems. Participants commonly connected this anxiety with the possibility that ovulatory dysfunction could make conception more difficult. The anxiety became more pronounced in accounts involving prolonged unsuccessful attempts to conceive. One participant explained:

*“I’ve been trying for two years, and I still haven’t been able to conceive… Every time I get my period, something inside me breaks”*

(P12)

A participant receiving trigger medication similarly linked her gradual loss of hope to learning that some menstrual cycles occurred without ovulation:

*“When I went to the doctor, I learned that I had periods when I wasn’t ovulating. From that moment on, I lost hope inside. I’m currently undergoing trigger medication treatment; with every failure, I start questioning myself. I’m doing everything I can, but I feel like it’s not enough.”*

(P11)

Another participant emphasized the cumulative psychological burden of repeated unsuccessful attempts:

*“We’ve been trying for three years, and I still haven’t been able to conceive. I’ve never even seen a single positive test. If only it happened once, so I could say it’s possible. A new obstacle comes up at every examination; it’s a very heavy psychological burden.”*

(P13)

#### 3.1.2. Emotional Impact of Diagnosis

For several participants, receiving the diagnosis or fertility-related information from a healthcare professional marked the beginning of subsequent anxiety. This effect was also evident among younger participants who were not currently planning a pregnancy, indicating that the emotional impact could precede active reproductive planning. For one participant, the way this information was communicated intensified her anxiety:

*“It’s already going to be hard for me to have a baby, maybe I won’t be able to have one at all. I’ll have to struggle a lot, it will be difficult. Maybe I’m infertile. This kind of anxiety was placed on me through that doctor’s information.”*

(P6)

Another participant described how the diagnosis created a sense of urgency about her reproductive future:

*“I was very frightened in that moment. I felt as if I had a time limit. Right now, this thought always stays in the back of my mind, wondering whether I’ll struggle in the future.”*

(P14)

#### 3.1.3. Initial Underestimation of the Condition

Some participants initially did not consider the potential effect of PMOS on fertility to be serious or believed that its effects were limited. This tendency was more apparent in accounts given before participants had experienced difficulty conceiving. One participant explained:

*“In PCOS, it just seems like not having periods. I don’t think there will be any problem with fertility”*

(P2)

Some participants reconsidered this initial perception after experiencing unsuccessful attempts to conceive. One participant reported that no fertility problem was initially identified during medical assessment; however, after two years without conception, she proceeded to in vitro fertilization treatment (P10).

### 3.2. Experiences and Evaluations Regarding Treatments

#### 3.2.1. Perception of Treatment as Temporary or Insufficient

Participants generally perceived current medical treatments as approaches that provided temporary relief rather than resolving the condition. This perception was particularly evident in their evaluations of hormonal contraceptives, whose effects were described as continuing only while the medication was used. One participant explained:

*“Birth control pills are just a temporary treatment method… I honestly haven’t seen an effective and permanent treatment”*

(P9)

Treatment experiences were also characterized by a recurring cycle of hope and disappointment. As one participant stated:

*“After every treatment, an expectation builds up, and then you’re devastated. Within this cycle, it even becomes hard to tell whether the treatments are working”*

(P13)

#### 3.2.2. Anger Toward the Weight-Focused Approach

Weight-focused recommendations were a source of frustration and self-blame, particularly when participants felt that their concerns were reduced to body weight. One participant reported that her condition had not improved despite losing 21 kg and described how repeated advice to lose weight led her to question whether she was responsible for the condition:

*“I think doctors put pressure on women. When they see a woman who is slightly overweight or a bit above her ideal weight, they immediately adopt the approach of ‘lose weight, then it will get better.’ This makes the woman feel even worse about herself. You start to think, ‘Did this happen because of me? Because of my weight?’”*

(P9)

#### 3.2.3. Hope and Positive Expectations Regarding Treatment

Despite negative treatment experiences, some participants maintained confidence in medical progress and the potential benefits of treatment. Awareness that PMOS was common and was receiving greater clinical attention appeared to partially ease uncertainty for these participants. One participant stated:

*“Medicine has advanced a lot. Since PCOS is now very common among women, I definitely think it’s being followed more closely and that there are better treatments.”*

(P5)

#### 3.2.4. Side Effects and Mistrust

Some participants described discontinuing treatment because of side effects or developing mistrust in its effectiveness. Medication was sometimes perceived as relieving one problem while creating another, which complicated continued engagement with treatment. One participant stated:

*“I went through a treatment process, but I had to stop because the side effects were too much. The medications fix one thing while breaking another.”*

(P3)

Another participant described the combined physical and emotional burden of treatment:

*“On one side there’s the physical burden, on the other an emotional collapse. It feels like the treatments will never end.”*

(P12)

### 3.3. Social Circle, Family, and Spousal Pressure

#### 3.3.1. Feeling of Inadequacy and Falling Behind

Participants described feelings of inadequacy and of being “left behind” when women around them became pregnant or had children. These feelings were particularly evident among participants actively attempting to conceive and were linked to socially defined expectations of motherhood and family completeness. One participant explained:

*“I always felt like half of myself… being a family meant… being together with a child”*

(P10)

However, feelings of inadequacy were not limited to participants who had attempted to conceive. One participant who had not yet attempted conception connected these feelings with societal expectations of motherhood:

*“I feel very inadequate. There are certain things this society imposes on us. Like becoming a mother.”*

(P8)

#### 3.3.2. Dilemma of Concealing or Disclosing the Diagnosis

Two different responses to disclosure were evident in participants’ accounts. Some concealed the diagnosis because they anticipated immediate pressure to conceive, whereas others disclosed it and subsequently experienced pressure from relatives. One participant explained that concealment reduced external pressure but could also feel isolating:

*“If I share it, the pressure of ‘then have a child right away’ starts immediately, I know that. Carrying this diagnosis inside me is sometimes very isolating.”*

(P14)

In contrast, another participant regretted disclosing her diagnosis because pressure subsequently came from both her own family and her spouse’s family:

*“A few weeks later, my mother-in-law started saying, ‘my dear, waiting isn’t good, try right away.’ My mother also pressures me by saying, ‘you need to act early.’ My husband is on my side, but carrying this pressure coming from both sides is very exhausting. Sometimes I regret having told them.”*

(P15)

#### 3.3.3. Resistance to Family and Social Pressure

Some participants actively resisted family and social expectations and emphasized their personal autonomy in reproductive decision-making. One participant stated:

*“There are expectations, but I don’t really care much. After all, it’s my life. I act according to my own opinions”*

(P3)

Another participant managed these expectations by preparing people around her for the possibility that conception could be difficult:

*“Whenever the subject comes up, I always try to lower expectations, and by lowering those expectations—if it gets hard, if we struggle, don’t come down on us, understand that—I think this way so that people’s expectations of me won’t be too high.”*

(P6)

#### 3.3.4. Support Received from Spouse

Most participants valued supportive and non-pressuring attitudes from their spouses. Spousal understanding was described as a source of support when participants were also managing expectations from their wider families. One participant stated:

*“My husband is always by my side. He even said that if it doesn’t happen, we don’t have to push it either. He doesn’t put any pressure on me about this.”*

(P10)

Another participant briefly emphasized her spouse’s understanding:

*“My husband knows, and he is very understanding.”*

(P14)

However, prolonged difficulty conceiving could also affect both partners. One participant explained:

*“My husband is very patient, thank God, but I can see the exhaustion in his eyes now too. We’re both worn out”*

(P13)

### 3.4. Expectations from Healthcare Professionals

#### 3.4.1. Need for Psychological Support

Nearly all participants expressed a need for psychological support, which they felt was absent from routine clinical care. They described a perceived emphasis on physical symptoms and test results, while the emotional burden of diagnosis and treatment remained largely unaddressed. One participant explained:

*“You break down emotionally during this process, and nobody tells you that”*

(P11)

Another participant emphasized that her emotional well-being had never been addressed during treatment:

*“Every time I go, only the test results are looked at, nobody asks how I feel. I’ve been in this process for three years, and not once has anyone asked, ‘this process is very hard, how are you?’ Psychological support is essential.”*

(P13)

#### 3.4.2. Need for Clear Information and a Roadmap

Participants wanted clear and individualized information after diagnosis rather than receiving a prescription alone. They expected healthcare professionals to explain their current condition, the steps that could be followed, possible future implications, and when further concern or intervention might be warranted. One participant stated:

*“I want a concrete roadmap like, ‘this is your current situation, we will follow these steps’”*

(P11)

Another participant wanted clearer information about the meaning and future implications of the diagnosis:

*“I would have wanted them to clearly explain what this diagnosis means, what steps could be taken in the future, and when I should be concerned. That way I could have felt more prepared.”*

(P14)

#### 3.4.3. Distrust in and Disappointment with the Healthcare System

Some participants, particularly those who had lived with PMOS for a longer period, described growing distrust and disappointment in the healthcare system. Repeated cycles of medication, testing, and weight-focused recommendations were perceived as providing limited guidance. One participant stated:

*“Honestly, I’ve lowered my expectations of health professionals to zero. That whole routine—period-inducing pills, go back and forth, give blood tests, now go lose some weight and come back—I don’t think any good guidance is being given at all.”*

(P6)

Another participant explained that this approach had discouraged her from seeking further care:

*“This approach makes a person feel inadequate, pressured, and labeled. That’s why I no longer want to go to the gynecology outpatient clinic”*

(P9)

#### 3.4.4. Need for a Multidisciplinary Team

Several participants emphasized that PMOS management should not be limited to consultations with a single clinician. They wanted coordinated support involving gynecology, nursing, dietetics, psychological care, and fertility services according to individual needs. One participant stated:

*“A doctor alone isn’t enough in this process; there needs to be a holistic support team”*

(P15)

Another participant similarly stated:

*“I would like this to be carried out systematically—a dietitian, a gynecologist, and, if necessary, an infertility specialist.”*

(P1)

### 3.5. Self-Management and Coping Strategies

#### 3.5.1. Nutrition and Exercise Adjustments

Almost all participants described making lifestyle changes intended to improve their chances of conceiving. Dietary adjustments included reducing sugar, gluten, and processed foods, while walking and Pilates were among the commonly described forms of exercise. One participant stated:

*“For two years I’ve been very careful about nutrition and exercise. I’ve cut down on sugar and gluten, and I do pilates regularly. I’ve also paid attention to my husband’s diet.”*

(P12)

Another participant described combining dietary changes, exercise, and stress management:

*“I try to eat a low-glycemic-index diet, and I do pilates and walking. I’ve also started paying attention to stress management, because I learned that stress negatively affects the cycle.”*

(P15)

#### 3.5.2. Use of Alternative and Herbal Approaches

Some participants reported using herbal teas, inositol, and other supplements alongside, or instead of, medical treatment. These approaches formed part of participants’ efforts to manage the condition outside routine medical treatment. One participant stated:

*“Instead of birth control pills, I started using herbal medicines and herbal teas as an alternative”*

(P10)

#### 3.5.3. Building Psychological Resilience

Several participants who had experienced prolonged difficulty conceiving described a change in perspective as a way of managing repeated disappointment and uncertainty. These strategies included reducing self-imposed pressure and viewing the process as a long-term journey rather than a competition. One participant stated:

*“I’m now trying to turn this process into a journey rather than a race”*

(P13)

Another participant described consciously avoiding persistent thoughts about conception:

*“I don’t want to fall into thoughts like ‘did it not happen again this month?’ every month. I try not to pressure myself too much and to let the process take its course”*

(P9)

#### 3.5.4. Reproductive Planning and Timing of Sexual Intercourse

Reproductive planning included deliberately timing sexual intercourse to increase the likelihood of conception. Although this represented an active effort to improve the chance of pregnancy, the absence of the expected outcome could intensify feelings of inadequacy. One participant explained:

*“In order to increase my chances of pregnancy, for two years I made sure to have intercourse with my husband every other day. But when pregnancy didn’t happen, I started to feel worse, thinking that despite doing all this I still couldn’t get pregnant. Over time, I began to feel inadequate and deficient”*

(P10)

## 4. Discussion

The present study found that fertility-related anxiety among women with PMOS was a multidimensional experience shaped by uncertainty about future conception, the emotional impact of diagnosis, treatment experiences, social expectations, and perceived gaps in professional support. Fertility-related anxiety varied according to participants’ individual circumstances, reproductive histories, relationships, and healthcare experiences. Responses to these experiences ranged from loss of hope and continued confidence in medical treatment to resistance to social pressure and the use of personal coping strategies. These findings demonstrate that fertility-related anxiety was not experienced uniformly but was influenced by participants’ individual, relational, and healthcare experiences.

Uncertainty about future fertility was a central concern, but its meaning and intensity varied according to reproductive history and personal circumstances. This finding is consistent with the study by Sarı and Güngör Satılmış [6], in which 72.9% of young adults with PMOS expressed concern about future infertility and 61.4% reported sometimes worrying that they would be unable to conceive. Findings from Özcan et al. [7] also showed that women diagnosed with PMOS frequently described anxiety, sadness, fear, and concern about infertility, and that uncertainty about childbearing was influenced by menstrual irregularity, information obtained online, social pressure, and communication with healthcare professionals. Holton et al. [5] further reported that women with PMOS were concerned about whether they could become pregnant and how they should prepare for pregnancy; some wanted fertility-related information at diagnosis or before actively considering pregnancy. Copp et al. [9] also reported that receiving a PMOS diagnosis could generate prolonged worry about infertility, including among women with relatively mild symptoms, and could create pressure to conceive earlier or alter life plans. Within our sample, the diagnosis was perceived by some women as imposing a time limit, whereas others initially underestimated its potential effect on fertility and revised that view after unsuccessful attempts to conceive. These contrasting experiences indicate the importance of providing balanced and individualized fertility information at the time of diagnosis.

Treatment was often perceived as temporary or insufficient, with unmet expectations producing recurring cycles of hope and disappointment. This pattern echoes Kim and Yi [23], whose participants described emotional exhaustion, frustration, and resignation when prolonged treatment and self-management efforts produced little perceptible improvement. Repeated weight-focused recommendations in our sample also contributed to frustration, self-blame, and distrust in healthcare. Consistent with this finding, Ismayilova and Yaya [24] reported that some women with PMOS felt that their symptoms and concerns were dismissed or attributed primarily to their weight. The 2023 International Evidence-based Guideline recognizes that women with PMOS may experience weight stigma in healthcare and recommends individualized, weight-inclusive practices that acknowledge the health benefits of lifestyle changes even in the absence of weight loss [3]. These findings do not diminish the clinical relevance of weight management; rather, they indicate that weight-related recommendations should be delivered within a broader, individualized care plan that includes clear explanations, ongoing support, and attention to women’s priorities.

The treatment narratives also illustrate the ambivalence of medicalized fertility care. Clinical monitoring and treatment offered hope and a pathway to action, but repeated testing, temporary symptom control, and weight-focused advice could also reorganize uncertainty into ongoing surveillance of bodies, cycles, and treatment outcomes. A recent qualitative study of women undergoing medically assisted reproduction described an internalized ‘clinical gaze’, fragmented body representations, and bodies experienced as repositories of reproductive hopes [25]. In the present sample, recruitment from a private clinic and participants’ fertility-treatment histories may likewise have intensified a treatment-centered framing of fertility and cycles of hope, disappointment, and self-scrutiny. This interpretation does not reject biomedical care; rather, it indicates that treatment should be accompanied by attention to how clinical routines shape embodied meaning, agency, and emotional burden.

Family and social expectations formed an important context for participants’ fertility-related anxiety. Accounts included feeling left behind when peers had children, managing whether to disclose the diagnosis, and experiencing pressure from family members to conceive, although some women actively resisted these expectations. Related concerns were identified by Sarı and Güngör Satılmış [6], whose study showed that young Turkish adults with PMOS worried about the condition’s possible effects on social and partner relationships. In a qualitative study involving Omani women with both PMOS and infertility, AlSumri et al. [26] reported strong expectations to conceive soon after marriage, pressure primarily from in-laws, and blame directed toward women for delayed conception. Most women in that study described receiving support from their partners, whereas marital tensions were more apparent when infertility had persisted for a longer period. In our sample, supportive and non-pressuring attitudes from spouses were valued, although prolonged difficulty conceiving could become emotionally exhausting for both partners. Because all participants in the Omani study had infertility, comparisons with the present sample should be interpreted cautiously. Nevertheless, these findings indicate that fertility-related anxiety is experienced within a broader network of family, marital, and sociocultural relationships.

The accounts of feeling inadequate, ‘left behind’, or incomplete can be interpreted as more than individual reactions to possible infertility. They suggest reproductive stigma: the anticipation or internalization of social judgments that position motherhood as a marker of feminine adulthood and family completeness. A recent qualitative meta-synthesis found that infertility-related stigma was shaped by social expectations surrounding motherhood and was associated with worthlessness, self-blame, isolation, and strained relationships [27]. A 2026 qualitative study similarly identified prescribed gender roles, responsibility and blame, and perceptions of infertility as failure of the female body as mechanisms of gendered infertility stigma [28]. These findings align with recent PCOS evidence that concerns about infertility can affect perceived femininity [12]. In the present study, concealment of the diagnosis and attempts to lower relatives’ expectations may therefore represent stigma-management strategies, whereas resistance to family pressure and supportive spousal responses show that participants also negotiated and contested these norms. Fertility-related anxiety should thus be understood not solely as fear of a biological outcome but also as anxiety about social recognition, gendered identity, and anticipated evaluation by others.

Expectations of healthcare professionals extended beyond medical treatment to psychological support, clear and individualized information, and coordinated multidisciplinary care. Kim and Yi [23] documented difficulties in self-management arising from insufficient or fragmented information, together with a need for understandable explanations, empathetic communication, and individualized guidance. Repeated encounters perceived as inadequate or predominantly weight-focused were also associated with distrust, and one woman described avoiding further gynecological care. This finding suggests that communication perceived as dismissive may weaken women’s engagement with healthcare services. The need for psychological support is also important in light of Zhang et al. [29], who found that greater fertility stress was associated with more depressive symptoms among women with PMOS, whereas greater psychological resilience was associated with fewer depressive symptoms. As that study was cross-sectional, these relationships should not be interpreted as causal. Nevertheless, the combined findings support routinely asking women about fertility-related distress and providing appropriate psychological referral when needed. Clear explanations and collaboration among gynecology, nursing, dietetics, fertility, and mental health professionals may help address the interconnected reproductive, metabolic, and psychological dimensions of PMOS.

Clinical communication appeared to function as a mechanism through which anxiety was either amplified or contained. Statements that implied inevitable infertility or reduced concerns to body weight may have been experienced as authoritative predictions, converting probabilistic risk into a perceived personal prognosis and reinforcing self-blame. Conversely, participants’ requests for clear explanations and a roadmap indicate that uncertainty may be more manageable when clinicians distinguish PMOS-associated risk from an individual diagnosis of infertility, acknowledge emotional responses, and discuss options consistent with reproductive intentions. A 2024 qualitative evidence synthesis found that PCOS healthcare interactions were especially challenging when medical information was not shared optimally, deliberation opportunities were insufficient, patient activation was unsupported, or system barriers were compounded by healthcare-professional behavior [30]. This supports interpreting timing, framing, shared deliberation, and relational validation as clinically consequential rather than incidental features of communication. Psychologically supportive, non-stigmatizing communication is therefore part of fertility care rather than an optional addition.

Self-management and coping encompassed dietary changes, exercise, herbal approaches, psychological reframing, and reproductive planning. This pattern is consistent with Kim and Yi [23], who found that women with PMOS relied on lifestyle changes, information seeking, and family support, although sustaining these efforts could become emotionally exhausting. The 2023 International Evidence-based Guideline recommends lifestyle intervention for all women with PMOS to improve metabolic health but emphasizes that management should be tailored to individual goals and preferences [3]. Herbal products were used alongside or instead of medical treatment. Because the study did not evaluate the content, safety, or effectiveness of these products, no conclusions can be drawn regarding their clinical value; however, their use highlights the importance of discussing complementary approaches openly and without judgment. For some women, psychological reframing reduced self-imposed pressure, whereas scheduling sexual intercourse specifically to achieve pregnancy intensified feelings of inadequacy when conception did not occur. Thus, self-management could foster a sense of agency while also becoming an additional source of emotional burden when efforts did not produce the expected outcome.

This study has several strengths. The study sample included participants with different symptom profiles, durations since diagnosis, and fertility histories, allowing for a range of experiences to be represented. Independent coding by three researchers, reflexive discussions, external expert review, and the use of direct quotations also supported the credibility and transparency of the findings. However, the study was conducted with 15 women recruited from a single private outpatient clinic in Istanbul, Türkiye, and the findings may not reflect the experiences of women from different healthcare settings or sociocultural backgrounds. All participants were married and childless, and nearly half had received fertility treatment; therefore, fertility-related concerns may have been particularly prominent in their accounts. This sample profile may have shaped not only the prominence but also the form of the reported anxiety. Restricting participation to married, childless women may have foregrounded narratives organized around anticipated motherhood, pressure to conceive after marriage, and perceptions of family completeness, while underrepresenting the experiences of women who are unmarried, already have children, do not intend to conceive, or understand parenthood differently. Recruitment from a single private clinic may also have selected women with access to private gynecological care and particular expectations of treatment and clinician communication; their experiences may differ from those of women using public services, receiving care in other regions, or not currently engaged in care. Accordingly, the findings are not intended to be statistically generalized, and their transferability should be assessed cautiously in relation to marital and parental status, reproductive intentions, treatment exposure, socioeconomic position, and healthcare context. As the findings were based on self-reported experiences, they may also have been influenced by recall or social desirability. The relatively focused interview guide and the interview duration of approximately 30–40 min may have limited the depth with which some experiences were explored. Although the interviews generated recurring patterns relevant to the study aim, their duration may have constrained extended narration, reflection on emotionally sensitive experiences, and exploration of changes over time or tensions within individual accounts. Some participants may also have required more time or repeated contact to articulate difficult fertility experiences. The analysis may therefore represent the principal dimensions of fertility-related anxiety more fully than the complexity and temporal development of each participant’s narrative. Future research using longer or repeated interviews, participant diaries, or longitudinal follow-up could provide greater narrative depth and clarify how anxiety changes across diagnostic and treatment trajectories. The interviews were conducted in the same clinical setting in which participants were receiving care. The interviewers were independent of the care team, participants were informed of this independence, and the interviews took place in a private room. Nevertheless, the institutional context may still have heightened perceived evaluation or encouraged cautious and socially desirable accounts of clinicians and services. Participants may therefore have moderated criticism or omitted experiences they considered sensitive, and the data may underrepresent the full intensity or range of concerns about healthcare-professional communication. Future qualitative studies could offer participants a choice of neutral, non-clinical, or remote interview locations and reinforce the separation between research participation and clinical care. Finally, although the English translations were reviewed for semantic and cultural equivalence, some nuances of the original Turkish accounts may not have been fully retained.

Future studies should include women from different healthcare settings, geographical regions, and sociocultural backgrounds to explore the transferability of these findings. Greater variation in age, parental status, reproductive intentions, and fertility-treatment experiences may provide a broader understanding of fertility-related anxiety in PMOS. Longitudinal designs could examine how these experiences change from diagnosis through different stages of reproductive planning and treatment. Including the perspectives of partners and healthcare professionals may also clarify how relational support and clinical communication shape women’s experiences. Intervention research is also needed to evaluate whether individualized fertility counselling, psychological support, and multidisciplinary care improve psychological and reproductive health outcomes.

## 5. Conclusions

Women with PMOS experienced fertility-related anxiety as a multidimensional phenomenon shaped by uncertainty about future conception, diagnosis and treatment experiences, social expectations, and interactions with healthcare professionals. These concerns varied according to women’s individual circumstances, reproductive histories, relationships, and healthcare experiences. Participants wanted clear and individualized fertility information, psychologically supportive communication, and coordinated care addressing the reproductive, metabolic, and emotional dimensions of PMOS. Healthcare professionals should therefore discuss fertility concerns from the time of diagnosis, assess fertility-related distress, and provide appropriate multidisciplinary support based on each woman’s needs and reproductive intentions.

## Figures and Tables

**Table 1:** Sociodemographic and Clinical Characteristics of the Participants (N = 15).

Participant	Age (Years)	Education	Employment	Duration Since Diagnosis (Years)	Previous Attempt to Conceive	Fertility Treatment	Effect on Thoughts About Having Children	Reported Symptoms
P1	26	Bachelor’s degree	Employed	7	Yes	No	Yes	MI, WG, A
P2	21	High school	Unemployed	2	No	No	No	MI, WG, H, A, IR
P3	34	Postgraduate	Unemployed	14	Yes	Yes	Yes	MI, WG, H, A
P4	22	Bachelor’s degree	Unemployed	1	No	No	Yes	MI, WG, H
P5	28	Bachelor’s degree	Unemployed	3	No	No	Yes	MI, H, IR
P6	32	Postgraduate	Employed	10	Yes	Yes	Yes	MI, WG, H, IR
P7	22	Bachelor’s degree	Unemployed	2	Yes	Yes	Yes	A
P8	21	High school	Unemployed	5	No	No	Yes	MI, H, A
P9	24	High school	Unemployed	4	Yes	No	Yes	MI, WG
P10	30	Postgraduate	Unemployed	2	Yes	Yes	Yes	A, WG
P11	24	Bachelor’s degree	Employed	5	Yes	Yes	Yes	MI, IR
P12	22	High school	Employed	1	Yes	Yes	Yes	MI
P13	28	Bachelor’s degree	Employed	9	Yes	Yes	Yes	MI, WG
P14	23	High school	Unemployed	5	No	No	Yes	-
P15	26	Bachelor’s degree	Employed	8	No	No	Yes	MI

Note: Participants could report more than one symptom. MI, menstrual irregularity; WG, weight gain; H, hirsutism; A, acne; IR, insulin resistance; -, no symptoms reported.

**Table 2:** Themes and Subthemes Derived from the Interviews with Women Diagnosed with PMOS.

Theme	Subthemes
Perceptions of the effect of PMOS on fertility	Anxiety and fear of the future Emotional impact of diagnosis Initial underestimation of the condition
Experiences and evaluations regarding treatments	Perception of treatment as temporary or insufficient Anger toward the weight-focused approach Hope and positive expectations regarding treatment Side effects and mistrust
Social circle, family, and spousal pressure	Feeling of inadequacy and “falling behind” Dilemma of concealing or disclosing the diagnosis Resistance to family and social pressure Support received from spouse
Expectations from healthcare professionals	Need for psychological support Need for clear information and a roadmap Distrust in and disappointment with the healthcare system Need for a multidisciplinary team
Self-management and coping strategies	Nutrition and exercise adjustments Use of alternative and herbal approaches Building psychological resilience Reproductive planning and timing of sexual intercourse

## Data Availability

The data supporting the findings of this study are available from the corresponding author upon reasonable request. The interview transcripts are not publicly available because they contain sensitive qualitative data and are subject to participant confidentiality and ethical restrictions.

## Supplementary Materials

The following supporting information can be downloaded at https://www.mdpi.com/article/10.3390/healthcare14193249/s1. Table S1: COREQ (Consolidated Criteria for Reporting Qualitative Research) 32-item checklist; Table S2: Semi-Structured Interview Guide; Table S3: Coding Tree for the Thematic Analysis.

## Abbreviations

The following abbreviations are used in this manuscript:

PMOS	Polyendocrine metabolic ovarian syndrome
PCOS	Polycystic ovary syndrome
COREQ	Consolidated Criteria for Reporting Qualitative Research

## References

[1] Teede H.J., Bahri Khomami M., Morman R., Laven J.S.E., Joham A.E., Costello M.F., Patil M., Rees D.A., Berry L., Cree M.G. (2026). Polyendocrine metabolic ovarian syndrome, the new name for polycystic ovary syndrome: A multistep global consensus process. Lancet.

[2] World Health Organization Polycystic Ovary Syndrome. https://www.who.int/news-room/fact-sheets/detail/polycystic-ovary-syndrome.

[3] Teede H.J., Tay C.T., Laven J.J.E., Dokras A., Moran L.J., Piltonen T.T., Costello M.F., Boivin J., Redman L.M., Boyle J.A. (2023). Recommendations from the 2023 international evidence-based guideline for the assessment and management of polycystic ovary syndrome. Eur. J. Endocrinol..

[4] Yin X., Ji Y., Chan C.L.W., Chan C.H.Y. (2021). The mental health of women with polycystic ovary syndrome: A systematic review and meta-analysis. Arch. Womens Ment. Health.

[5] Holton S., Hammarberg K., Johnson L. (2018). Fertility concerns and related information needs and preferences of women with PCOS. Hum. Reprod. Open.

[6] Sarı E., Güngör Satılmış İ. (2024). Concerns about fertility, social appearance, and relationships in young adults with polycystic ovary syndrome. J. Reprod. Infant Psychol..

[7] Özcan H., Altın S., Şekercioğlu E., Köse S., Güzel E. (2025). Concerns of women diagnosed with polycystic ovary syndrome regarding the process and having children: A qualitative study. Int. J. Clin. Pract..

[8] Hassan M.M., Lim K.B., Yeo S.F., Ameeq M. (2025). Cultural perceptions and emotional well-being among married women with polycystic ovary syndrome experiencing fertility difficulties in Southern Punjab, Pakistan: A cross-sectional study. Healthcare.

[9] Copp T., Hersch J., Muscat D.M., McCaffery K.J., Doust J., Dokras A., Mol B.W., Jansen J. (2019). The benefits and harms of receiving a polycystic ovary syndrome diagnosis: A qualitative study of women’s experiences. Hum. Reprod. Open.

[10] Tomlinson J., Pinkney J., Adams L., Stenhouse E., Bendall A., Corrigan O., Letherby G. (2017). The diagnosis and lived experience of polycystic ovary syndrome: A qualitative study. J. Adv. Nurs..

[11] Hadjiconstantinou M., Mani H., Patel N., Levy M., Davies M., Khunti K., Stone M. (2017). Understanding and supporting women with polycystic ovary syndrome: A qualitative study in an ethnically diverse UK sample. Endocr. Connect..

[12] Elghobashy M., Lau G.M., Davitadze M., Gillett C.D.T., O’Reilly M.W., Arlt W., Lindenmeyer A., Kempegowda P., PCOS SEva Working Group (2023). Concerns and expectations in women with polycystic ovary syndrome vary across age and ethnicity: Findings from PCOS Pearls Study. Front. Endocrinol..

[13] Sandelowski M. (2000). Whatever happened to qualitative description?. Res. Nurs. Health.

[14] Tong A., Sainsbury P., Craig J. (2007). Consolidated criteria for reporting qualitative research (COREQ): A 32-item checklist for interviews and focus groups. Int. J. Qual. Health Care.

[15] Saunders B., Sim J., Kingstone T., Baker S., Waterfield J., Bartlam B., Burroughs H., Jinks C. (2018). Saturation in qualitative research: Exploring its conceptualization and operationalization. Qual. Quant..

[16] Gibson-Helm M., Teede H., Dunaif A., Dokras A. (2017). Delayed diagnosis and a lack of information associated with dissatisfaction in women with polycystic ovary syndrome. J. Clin. Endocrinol. Metab..

[17] Snyder B.S. (2006). The lived experience of women diagnosed with polycystic ovary syndrome. J. Obstet. Gynecol. Neonatal Nurs..

[18] Braun V., Clarke V. (2006). Using thematic analysis in psychology. Qual. Res. Psychol..

[19] Braun V., Clarke V. (2021). Can I use TA? Should I use TA? Should I not use TA? Comparing reflexive thematic analysis and other pattern-based qualitative analytic approaches. Couns. Psychother. Res..

[20] Lincoln Y.S., Guba E.G. (1985). Naturalistic Inquiry.

[21] Korstjens I., Moser A. (2018). Series: Practical guidance to qualitative research. Part 4: Trustworthiness and publishing. Eur. J. Gen. Pract..

[22] Berger R. (2015). Now I see it, now I don’t: Researcher’s position and reflexivity in qualitative research. Qual. Res..

[23] Kim M., Yi S.J. (2025). Exploring the disease experience in women with PCOS: A qualitative content analysis. Healthcare.

[24] Ismayilova M., Yaya S. (2022). “I felt like she didn’t take me seriously”: A multi-methods study examining patient satisfaction and experiences with polycystic ovary syndrome in Canada. BMC Women’s Health.

[25] Fusco C., Masaro C., Calvo V. (2024). ‘I feel like I was born for something that my body can’t do’: A qualitative study on women’s bodies within medicalized infertility in Italy. Psychol. Health.

[26] AlSumri H., Szatkowski L., Gibson J., Fiaschi L., Bains M. (2023). Psychosocial impacts of infertility among Omani women with polycystic ovarian syndrome: A qualitative study. Int. J. Fertil. Steril..

[27] Lee J., Kim S., Nam S.-H. (2025). ‘Living with silence and shame’: A meta-synthesis of women’s lived experiences of infertility-related stigma. Int. J. Womens Health.

[28] Bornstein M., Church A.C., Masterson K., Gipson J.D., Norris A.H. (2026). ‘What’s wrong with me?’: Women’s perceptions of gendered infertility stigma. Soc. Sci. Med..

[29] Zhang Z., Liu M., Zhao F., Chen H., Chen X. (2024). Fertility stress, psychological resilience, and depressive symptoms in women with polycystic ovary syndrome. Cureus.

[30] Harrison C., Tay C.T., Busby M.A., Mousa A., Teede H., Boivin J. (2024). Challenges experienced by those with polycystic ovary syndrome in healthcare interactions: A qualitative evidence synthesis. Reprod. Biomed. Online.

