# Supplementary material for: Experiences of Fertility-Related Anxiety Among Women with Polyendocrine Metabolic Ovarian Syndrome: A Qualitative Study
Source: Healthcare (Basel). 2026 Oct 1;14(19):3249. doi: 10.3390/healthcare14193249 (PMC13665244; doi:10.3390/healthcare14193249)
Supplement: Supplementary file 1 [file healthcare-14-03249-s001.zip › Supplementary Table S1 Healthcare COREQ FINAL.pdf]

Supplementary Table S1

COREQ (Consolidated Criteria for Reporting Qualitative Research) 32-item checklist

Reference: Tong A, Sainsbury P, Craig J. Consolidated criteria for reporting qualitative research (COREQ): a 32-item checklist for interviews and focus groups. Int J Qual Health Care 2007;19:349–357.

Manuscript: Experiences of Fertility-Related Anxiety among Women with Polyendocrine Metabolic Ovarian Syndrome: A Qualitative Study

No.	Item	Guide question / description	Reported on
	**Domain 1: Research team and reflexivity**		
1	Interviewer/facilitator	Which author(s) conducted the interview?	Materials and Methods—Data Collection
2	Credentials	What were the researcher's credentials?	Materials and Methods—Researcher Characteristics and Reflexivity (two researchers held MSc degrees and one researcher held a PhD).
3	Occupation	What was their occupation at the time of the study?	Materials and Methods—Data Collection (university-based academic researchers with clinical experience in women’s health).
4	Gender	Was the researcher male or female?	Materials and Methods—Researcher Characteristics and Reflexivity (all three researchers were female).
5	Experience and training	What experience or training did the researcher have?	Materials and Methods—Researcher Characteristics and Reflexivity.
6	Relationship established	Was a relationship established prior to study commencement?	Materials and Methods—Data Collection (no prior relationship)

No.	Item	Guide question / description	Reported on
7	Participant knowledge of the interviewer	What did participants know about the researcher?	Materials and Methods—Data Collection (participants were informed that the interviewers were academic researchers conducting research independently of their clinical care).
8	Interviewer characteristics	What characteristics were reported about the interviewer/facilitator?	Materials and Methods—Researcher Characteristics and Reflexivity.
	**Domain 2: Study design**		
9	Methodological orientation and theory	What methodological orientation was stated?	Materials and Methods—Study Design and Data Analysis (qualitative descriptive design; codebook approach to thematic analysis).
10	Sampling	How were participants selected?	Materials and Methods—Participants and Setting (purposive sampling)
11	Method of approach	How were participants approached?	Materials and Methods—Participants and Setting (participants were approached face-to-face by the researchers).
12	Sample size	How many participants were included?	Materials and Methods—Participants and Setting; Results (N=15)
13	Non-participation	How many refused or dropped out?	Materials and Methods—Participants and Setting (no refusals or withdrawals occurred).
14	Setting of data collection	Where was the data collected?	Materials and Methods—Data Collection (private clinic room)

No.	Item	Guide question / description	Reported on
15	Presence of non-participants	Was anyone else present besides participants and researchers?	Materials and Methods—Data Collection (no one else present)
16	Description of sample	What are the important characteristics of the sample?	Results—Table 1
17	Interview guide	Were questions, prompts, and guides provided?	Materials and Methods—Data Collection (pilot-tested guide); Supplementary Table S2 (full interview guide).
18	Repeat interviews	Were repeat interviews carried out?	Materials and Methods—Data Collection (each participant completed one interview; no repeat interviews were conducted).
19	Audio/visual recording	Did the research use audio or visual recording?	Materials and Methods—Data Collection (audio-recorded)
20	Field notes	Were field notes made during and/or after the interview?	Materials and Methods—Data Collection (no field notes were taken).
21	Duration	What was the duration of the interviews?	Materials and Methods—Data Collection (30–40 minutes)
22	Data saturation	Was data saturation discussed?	Materials and Methods—Participants and Setting (saturation after 15 interviews)
23	Transcripts returned	Were transcripts returned to participants for comment?	Materials and Methods—Data Collection (transcripts were not returned to participants for comment or correction).
	**Domain 3: Analysis and findings**		
24	Number of data coders	How many coders coded the data?	Materials and Methods—Data Analysis (three researchers).

No.	Item	Guide question / description	Reported on
25	Description of the coding tree	Did authors provide a description of the coding tree?	Materials and Methods—Data Analysis; Results—Table 2; Supplementary Table S3 (complete coding tree showing initial codes, intermediate categories, subthemes, and final themes).
26	Derivation of themes	Were themes identified in advance or derived from the data?	Materials and Methods—Data Analysis (themes were derived inductively from the data).
27	Software	What software was used to manage the data?	Materials and Methods—Data Analysis (coding was performed manually without qualitative data analysis software).
28	Participant checking	Did participants provide feedback on the findings?	Materials and Methods—Trustworthiness (participant checking was not undertaken).
29	Quotations presented	Were participant quotations used to illustrate themes?	Results—Themes 1–5
30	Data and findings consistent	Was there consistency between the data and the findings?	Results—Themes 1–5
31	Clarity of major themes	Were major themes clearly presented?	Results—five themes presented with Table 2
32	Clarity of minor themes	Is there a description of diverse cases or minor themes?	Results—Table 2 (subthemes); contrasting participant accounts within each theme
