# Supplementary material for: Experiences of Fertility-Related Anxiety Among Women with Polyendocrine Metabolic Ovarian Syndrome: A Qualitative Study
Source: Healthcare (Basel). 2026 Oct 1;14(19):3249. doi: 10.3390/healthcare14193249 (PMC13665244; doi:10.3390/healthcare14193249)
Supplement: Supplementary file 1 [file healthcare-14-03249-s001.zip › Supplementary Table S2 final.pdf]

Supplementary Table S2. Semi-Structured Interview Guide

No.	Interview question
1	Could you describe what being diagnosed with PCOS means to you and how, if at all, it has influenced your thoughts or feelings about fertility?
2	Could you describe your experiences with the healthcare, treatments, or recommendations you have received for PCOS?
3	How, if at all, have your spouse, family members, or wider social environment influenced your experiences related to PCOS and fertility?
4	Could you describe any information, communication, or support you have received from healthcare professionals and whether it met your needs?
5	Have you used any personal strategies to manage PCOS or fertility-related thoughts and feelings? If so, could you describe them?

The following open-ended questions were used to guide the individual interviews. Probing questions were used when clarification or elaboration was needed.

Note: *Because the term "PMOS" had only recently been introduced at the time of data collection and was not yet widely familiar to participants, the established term "PCOS" was used in the original interview guide to ensure clarity and has therefore been retained in this supplementary table.*
