# Supplementary material for: Experiences of Fertility-Related Anxiety Among Women with Polyendocrine Metabolic Ovarian Syndrome: A Qualitative Study
Source: Healthcare (Basel). 2026 Oct 1;14(19):3249. doi: 10.3390/healthcare14193249 (PMC13665244; doi:10.3390/healthcare14193249)
Supplement: Supplementary file 1 [file healthcare-14-03249-s001.zip › Supplementary Table S3 Coding Tree.pdf]

Supplementary Table S3

Coding Tree for the Thematic Analysis

The coding tree shows how data-near initial codes were grouped into intermediate categories, refined into subthemes, and synthesized into the five final themes. Initial codes are presented in concise English wording corresponding to the meanings expressed in the reported participant accounts.

Final theme	Subtheme	Intermediate categories	Initial codes
**Perceptions of the effect of PMOS on fertility**	**Anxiety and fear of the future**	• Anticipated infertility and uncertainty<br>• Cumulative distress following unsuccessful attempts	• Fear of being unable to conceive<br>• Linking anovulation with difficulty conceiving<br>• Losing hope after learning that some cycles were anovulatory<br>• Menstruation as a recurring sign of disappointment<br>• Questioning oneself after treatment failure<br>• Psychological burden of repeated negative pregnancy tests
**Perceptions of the effect of PMOS on fertility**	**Emotional impact of diagnosis**	• Diagnosis-triggered perception of reproductive threat<br>• Perceived reproductive time pressure<br>• Anxiety intensified by clinical communication	• Diagnosis as the beginning of fertility anxiety<br>• Being told that conception may be difficult<br>• Imagining the possibility of infertility<br>• Feeling frightened after diagnosis<br>• Perceiving a limited time for childbearing<br>• Worrying about fertility before actively planning pregnancy
**Perceptions of the effect of PMOS on fertility**	**Initial underestimation of the condition**	• Minimizing the reproductive meaning of PMOS<br>• Reappraising PMOS after difficulty conceiving	• Viewing PMOS mainly as menstrual irregularity<br>• Expecting no fertility problem<br>• Underestimating the condition before attempting conception<br>• Reconsidering the condition after unsuccessful attempts<br>• Proceeding to in vitro fertilization after prolonged non-conception
**Experiences and evaluations regarding treatments**	**Perception of treatment as temporary or insufficient**	• Symptom control without perceived resolution<br>• Cycle of treatment-related hope and disappointment	• Viewing hormonal contraceptives as temporary<br>• Perceiving no effective or permanent treatment<br>• Building expectations after each treatment<br>• Feeling devastated when treatment does not produce the expected result<br>• Uncertainty about whether treatment is working
**Experiences and evaluations regarding treatments**	**Anger toward the weight-focused approach**	• Reduction of care to body weight<br>• Internalized blame and emotional harm	• Feeling that concerns are reduced to weight<br>• Receiving repeated advice to lose weight<br>• Seeing no improvement despite substantial weight loss<br>• Feeling pressured by clinicians<br>• Questioning whether the condition is one's own fault
**Experiences and evaluations regarding treatments**	**Hope and positive expectations regarding treatment**	• Confidence in medical progress<br>• Reassurance from recognition of PMOS as common	• Believing that medicine has advanced<br>• Believing that PMOS is followed more closely because it is common<br>• Expecting improved treatment options<br>• Maintaining hope in treatment despite uncertainty
**Experiences and evaluations regarding treatments**	**Side effects and mistrust**	• Adverse effects undermining treatment continuation<br>• Accumulating physical and emotional treatment burden	• Stopping medication because of side effects<br>• Perceiving that medication solves one problem while creating another<br>• Experiencing simultaneous physical and emotional burden<br>• Feeling that treatment will never end<br>• Mistrusting treatment effectiveness

Final theme	Subtheme	Intermediate categories	Initial codes
**Social circle, family, and spousal pressure**	**Feeling of inadequacy and falling behind**	• Motherhood and family-completeness norms<br>• Social comparison and perceived delay	• Feeling incomplete without a child<br>• Defining family as including a child<br>• Feeling inadequate<br>• Experiencing motherhood as a social expectation<br>• Feeling left behind when peers become pregnant or have children
**Social circle, family, and spousal pressure**	**Dilemma of concealing or disclosing the diagnosis**	• Anticipated pressure and concealment-related isolation<br>• Disclosure followed by pressure and regret	• Hiding the diagnosis to avoid immediate pressure to conceive<br>• Carrying the diagnosis alone<br>• Feeling isolated by concealment<br>• Experiencing pressure from one's own family and in-laws<br>• Regretting disclosure
**Social circle, family, and spousal pressure**	**Resistance to family and social pressure**	• Protecting reproductive autonomy<br>• Managing other people's expectations	• Emphasizing that reproductive decisions are personal<br>• Disregarding external expectations<br>• Lowering relatives' expectations<br>• Preparing family members for possible difficulty conceiving<br>• Asking others not to blame or pressure the couple
**Social circle, family, and spousal pressure**	**Support received from spouse**	• Non-pressuring spousal support<br>• Shared emotional burden within the couple	• Having a spouse who remains supportive<br>• Receiving acceptance if pregnancy does not occur<br>• Feeling understood by one's spouse<br>• Experiencing the spouse as a buffer against family pressure<br>• Recognizing the spouse's exhaustion after prolonged difficulty conceiving
**Expectations from healthcare professionals**	**Need for psychological support**	• Emotional needs left unaddressed<br>• Predominantly biomedical clinical encounters	• Breaking down emotionally during the process<br>• Receiving attention to test results but not feelings<br>• Never being asked about emotional well-being<br>• Viewing psychological support as essential<br>• Perceiving psychological care as absent from routine services
**Expectations from healthcare professionals**	**Need for clear information and a roadmap**	• Need for individualized explanation<br>• Need for anticipatory guidance and follow-up planning	• Wanting an explanation of the current situation<br>• Wanting clearly sequenced next steps<br>• Wanting the diagnosis explained in understandable terms<br>• Wanting information about future implications<br>• Wanting to know when concern or intervention is warranted<br>• Feeling more prepared through clear guidance
**Expectations from healthcare professionals**	**Distrust in and disappointment with the healthcare system**	• Repetitive and fragmented care<br>• Disengagement following stigmatizing encounters	• Lowering expectations of healthcare professionals<br>• Experiencing a repeated medication-test-weight-loss cycle<br>• Perceiving an absence of meaningful guidance<br>• Feeling inadequate, pressured, or labeled in care<br>• Avoiding further gynecological care
**Expectations from healthcare professionals**	**Need for a multidisciplinary team**	• Preference for coordinated holistic care<br>• Need for profession-specific support	• Believing that one physician is insufficient<br>• Wanting a holistic support team<br>• Wanting coordinated dietetic and gynecological care<br>• Wanting infertility expertise when needed<br>• Wanting psychological and nursing support according to individual need

Final theme	Subtheme	Intermediate categories	Initial codes
**Self-management and coping strategies**	**Nutrition and exercise adjustments**	• Dietary self-management<br>• Physical activity and broader lifestyle regulation	• Reducing sugar and gluten<br>• Choosing a low-glycemic-index diet<br>• Reducing processed foods<br>• Doing pilates and walking regularly<br>• Managing stress to support cycle regulation<br>• Attending to the spouse's diet
**Self-management and coping strategies**	**Use of alternative and herbal approaches**	• Use of complementary products<br>• Self-directed alternatives to routine medication	• Using herbal medicines<br>• Drinking herbal teas<br>• Using inositol or other supplements<br>• Using complementary approaches alongside medical treatment<br>• Replacing hormonal medication with alternative approaches
**Self-management and coping strategies**	**Building psychological resilience**	• Cognitive reframing of the fertility process<br>• Reducing self-imposed pressure	• Viewing the process as a journey rather than a race<br>• Avoiding monthly rumination about conception<br>• Allowing the process to take its course<br>• Reducing pressure placed on oneself<br>• Adapting to repeated disappointment and uncertainty
**Self-management and coping strategies**	**Reproductive planning and timing of sexual intercourse**	• Active timing to increase the chance of conception<br>• Effort-related distress when pregnancy does not occur	• Scheduling intercourse every other day<br>• Timing intercourse to increase pregnancy likelihood<br>• Persisting with reproductive planning over time<br>• Feeling worse when pregnancy does not occur despite sustained effort<br>• Feeling inadequate or deficient

Note: PMOS, polyendocrine metabolic ovarian syndrome. Coding was conducted manually. The initial codes shown here are data-near English formulations aligned with the reported participant accounts and final analytic structure; related codes were compared across the dataset and consolidated through researcher consensus.
